# Genome-Wide Adductomics Analysis Reveals Heterogeneity in the Induction and Loss of Cyclobutane Thymine Dimers across Both the Nuclear and Mitochondrial Genomes

**DOI:** 10.3390/ijms20205112

**Published:** 2019-10-15

**Authors:** Alaa S. Alhegaili, Yunhee Ji, Nicolas Sylvius, Matthew J. Blades, Mahsa Karbaschi, Helen G. Tempest, George D. D. Jones, Marcus S. Cooke

**Affiliations:** 1 Oxidative Stress Group, University of Leicester, Leicester LE1 9HN, UKmkarbasc@fiu.edu (M.K.); 2 Radiobiology & DNA Damage Group, Leicester Cancer Research Centre, University of Leicester, Leicester LE1 9HN, UK; 3 Present Addresses: Department of Medical Laboratory Sciences, Prince Sattam bin Abdulaziz University, P.O. Box 422, Alkharj 11942, Kingdom of Saudi Arabia; 4 Present Addresses: Oxidative Stress Group, Department of Environmental Health Sciences, Florida International University, Miami, FL 33199, USA; yji008@fiu.edu; 5 NUCLEUS Genomics, Core Biotechnology Services, University of Leicester, Leicester LE1 9HN, UK; ns249@leicester.ac.uk; 6 Bioinformatics and Biostatistics Analysis Support Hub (BBASH), Core Biotechnology Services, University of Leicester, Leicester LE1 9HN, UK; mb492@leicester.ac.uk; 7 Present Addresses: Department of Human Molecular Genetics, Herbert Wertheim College of Medicine, Florida International University, Miami, FL 33199, USA; htempest@fiu.edu; 8 Present Addresses: Biomolecular Sciences Institute, Florida International University, Miami, FL 33199, USA; 9 Department of Genetics, University of Leicester, Leicester LE1 9HN, UK

**Keywords:** DNA damage, DNA repair, UV radiation, genome analysis, next generation sequencing

## Abstract

The distribution of DNA damage and repair is considered to occur heterogeneously across the genome. However, commonly available techniques, such as the alkaline comet assay or HPLC-MS/MS, measure global genome levels of DNA damage, and do not reflect potentially significant events occurring at the gene/sequence-specific level, in the nuclear or mitochondrial genomes. We developed a method, which comprises a combination of Damaged DNA Immunoprecipitation and next generation sequencing (DDIP-seq), to assess the induction and repair of DNA damage induced by 0.1 J/cm^2^ solar-simulated radiation at the sequence-specific level, across both the entire nuclear and mitochondrial genomes. DDIP-seq generated a genome-wide, high-resolution map of cyclobutane thymine dimer (T<>T) location and intensity. In addition to being a straightforward approach, our results demonstrated a clear differential distribution of T<>T induction and loss, across both the nuclear and mitochondrial genomes. For nuclear DNA, this differential distribution existed at both the sequence and chromosome level. Levels of T<>T were much higher in the mitochondrial DNA, compared to nuclear DNA, and decreased with time, confirmed by qPCR, despite no reported mechanisms for their repair in this organelle. These data indicate the existence of regions of sensitivity and resistance to damage formation, together with regions that are fully repaired, and those for which > 90% of damage remains, after 24 h. This approach offers a simple, yet more detailed approach to studying cellular DNA damage and repair, which will aid our understanding of the link between DNA damage and disease.

## 1. Introduction

DNA damage arises from endogenous sources, such as normal cellular metabolism, or exogenous sources, including exposure to ultraviolet radiation (UVR), chemicals and ionising radiation. These damaging agents modify the structure of DNA components, which may subsequently lead to mutations or cell death, with implication for diseases such as cancer. Arguably, the most serious effects of UVR exposure are DNA mutation, and carcinogenesis [1]. The absorption of UVR leads to the induction of damage to DNA by forming photoproducts, such as cyclobutane pyrimidine dimers (CPD), that are mutagenic, and therefore potentially carcinogenic [2]. Additionally, an indirect mechanism, via the chemoexcitation of melanin, has been reported to induce CPD [3]. Protecting the cell from the detrimental effects of damage are a variety of DNA repair pathways, of which nucleotide excision repair is of greatest importance for the removal of CPD [4,5].

The induction of damage by UVR depends upon the DNA sequence, local structure and chromatin environment/organisation [6,7] which, in part, contributes to the expected, differential distribution of damage formation. The other major factor determining the distribution of damage is DNA repair, which is mediated by distinct DNA repair pathways, and here chromatin organisation also plays a role [8], as chromatin structure and accessibility alters following UVR exposure [9]. These repair pathways are critical to maintain the integrity of the genome and prevent disease [10]. Furthermore, there is evidence that cells prioritise repair machinery to regions of specific need, to minimise disruption of function. For example, it is well established that repair is site-specific, with preferential removal of DNA damage from transcriptionally active genes over inactive regions [11,12] and transcribed strand-specific repair [12]. Furthermore, the nature of the lesion influences whether or not there is preferential repair in transcriptionally active genes, and in what stage of the cell cycle repair occurs [13]. Even within genes, particular regions may be favoured, such as the 5′ portion of the *DHFR* gene [14], although the molecular basis for such differential repair across genes remains subject to speculation. Supporting the importance of sequence-specific damage formation and repair is evidence that hotspots of CPD persistence are more likely to yield mutations [15,16]; about 80–90% of all human cancers can be correlated to regions of unrepaired DNA [17]; and that in melanoma, changes to local DNA structure favour the formation of CPD hotspots, which are highly correlated with sites of recurrent mutation [18].

A growing number of techniques evaluating damage and repair within discrete locations are now emerging. Initially, this was targeted towards individual genes, e.g., through the use of ligation-mediated PCR [19] and immuno-coupled PCR [20]. However, more recently, genome-wide mapping of damage has become possible (reviewed in Mao et al. [18]). The earliest reports were limited to providing information at a chromosomal level only, with rather crude resolution [21] or offering little information in terms of gene-specific or intergenic regions [22]. In the last few years there has been a small number of reports in the literature describing methods for the genome-wide mapping of different types of DNA damage at high resolution. These methods include a series of approaches based upon combinations of excision repair enzymes (e.g., the Excision-seq approach [23]), modifications of methodology to map ribonucleotide incorporation [24], or damaged DNA immunoprecipitation (DDIP—analagous to methylated DNA immunoprecipitation (MeDIP) [25], coupled with microarray (DDIP-chip, e.g., Teng et al. [26]) or next generation sequencing (DDIP-seq),. These have then been applied to study the formation of a variety of DNA damage products e.g., CPD [7], (6-4) photoproducts [27], platinum-induced guanine adducts [28], double-strand breaks [29], 8-oxo-7,8-dihydro-2′-deoxyguanosine (8-oxodG) [25,30], and uracil [23]. Whilst DDIP-chip is a sensitive, reliable assay for DNA damage, and can evaluate the location of DNA damage at high resolution (100–1000 bp), this approach does preclude detection at specific sites for which there is not array coverage. Additionally, to cover the entire human genome by microarray with high resolution, the use of multiple microarrays is required, which may not be practical, or financially feasible [31].

Here, we report the application of a straightforward method that utilises the DDIP-seq approach to analyse UVR-induced DNA damage and repair across the entire human genome. DDIP-seq was used to characterise solar simulated radiation (SSR)-induced DNA damage and repair in the genome of human skin keratinocytes, and adds to our growing understanding of the distribution of damage and repair in both the nuclear and mitochondrial genomes. 

## 2. Results

### 2.1. The Effect of SSR Irradiation on HaCaT Cell Viability

**Figure 1:**
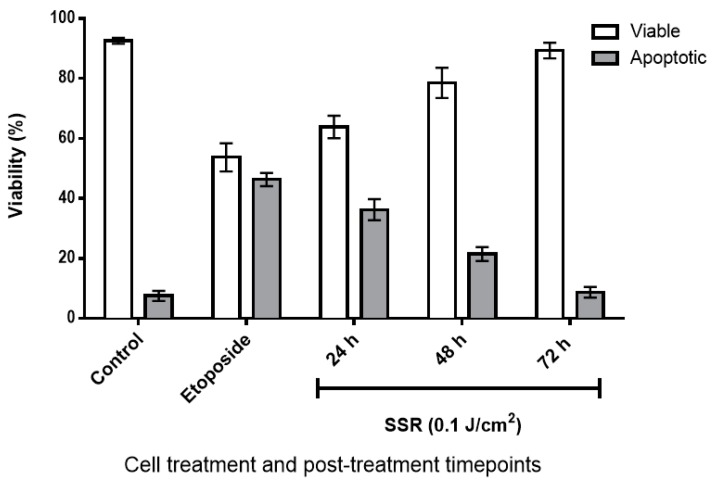
Cell viability determined by Annexin V staining and flow cytometry. HaCaT cells were irradiated with 0.1 J/cm^2^ SSR and then stained with Annexin V and propidium iodide, and assayed by flow cytometry after 24, 48 and 72 h. Control cells were sham-irradiated. Etoposide was used as a positive control, and viability assayed immediately after exposure. Error bars represent the mean ± SEM for three independent experiments.

Following the exposure to 0.1 J/cm^2^ of SSR, the cells were allowed to recover for 24, 48 and 72 h. The administered dose of SSR did induce some cell death, however, most cells were viable and capable of repair and growth (Figure 1). The dose of SSR used is considered to be in the range of the erythemal dose (0.1 J/cm^2^–0.2 J/cm^2^) in Europe, according to the Tropospheric Emission Monitoring Internet Service (TEMIS).

### 2.2. Optimisation of DNA:Anti-T<>T MAb Ratio

**Figure 2:**
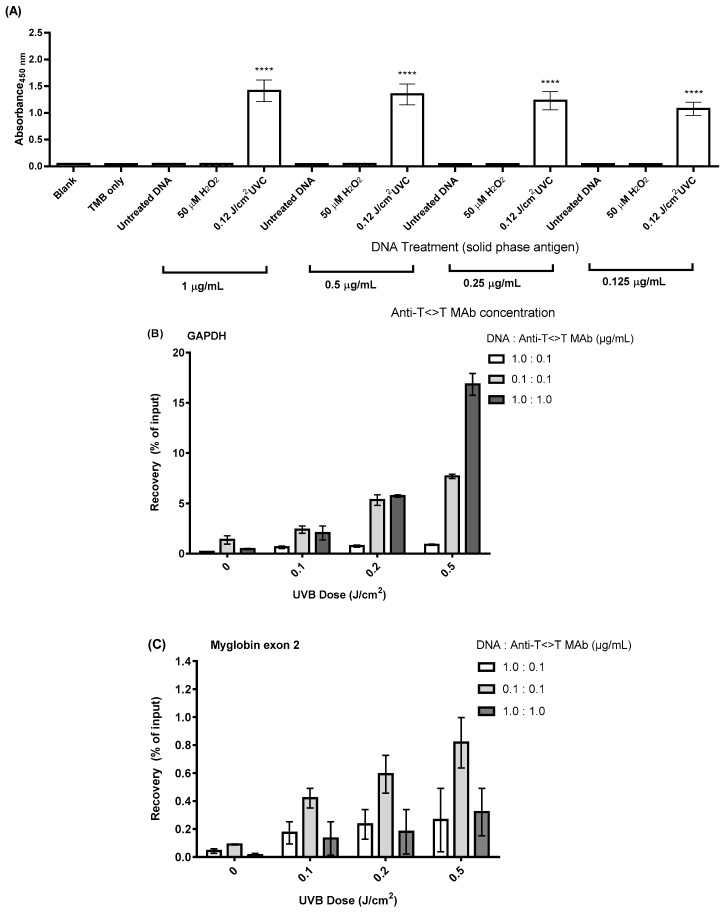
Specificity of the anti-T<>T Mab, and its optimisation, determined by DDIP-qPCR. DNA immunoprecipitation was performed using the MeDIP kit from Diagenode, with commercially available, extracted, human DNA irradiated with 0, 0.1, 0.2 and 0.5 J/cm^2^ UVB, and a monoclonal antibody against thymine dimers (T<>T). (**A**) ELISA results demonstrating the specificity of the anti-T<>T Mab for UV-modified DNA. Quantitative PCR was performed using primers specific for (**B**) the *GAPDH* gene promoter, an actively expressed gene, and (**C**) the *Myoglobin* exon 2, an inactive gene. Recovery was expressed as a percentage of the amount of immunoprecipitated DNA compared to the input DNA after qPCR. The results are presented as the mean ± SEM of three independent experiments. **** represents *p* < 0.001.

We based our protocol for DDIP-Seq upon a commercially available MeDIP assay, with optimisation for the detection of T<>T. In addition to the manufacturer’s information for the anti-T<>T MAb, and previous use [27], we provided some additional characterisation data. These data demonstrated that the anti-T<>T MAb overwhelmingly recognised UVC-induced modified DNA, the predominant lesion in which is T<>T, over unmodified or H_2_O_2_-modified DNA (Figure 2A).

For initial method development, we used commercially available human, genomic DNA, which was irradiated with UVB (0, 0.1, 0.2 and 0.5 J/cm^2^), fragmented to 100–300 bp by sonication, and the DNA:antibody ratio varied. After the immunoprecipitation, the samples were quantified by qPCR (DDIP-qPCR) at the *GAPDH* promoter and *Myoglobin* exon 2 regions, as representative transcriptionally active, and inactive genes, respectively. The most pronounced dose-response was seen with a DNA/antibody ratio of 1:1 μg/mL, for *GAPDH* (Figure 2B), although this was less pronounced for *Myoglobin* exon 2 (Figure 2C). 

### 2.3. DDIP-qPCR Quantification of the Induction and Repair of CPDs Induced by UVR

**Figure 3:**
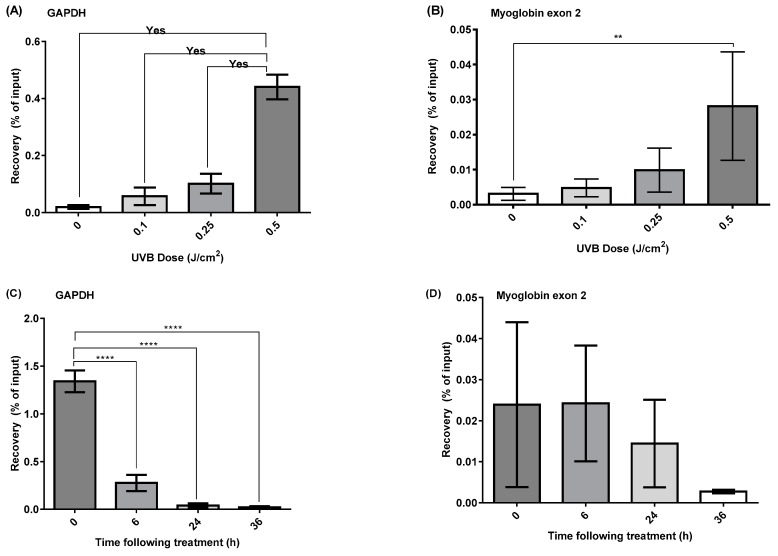
DDIP-qPCR analysis of the induction, and repair, of T<>T in nuclear DNA of HaCaT cells exposed to UVR. DDIP-qPCR for T<>T was performed immediately after exposure using primers specific for the (**A**) *GAPDH* gene promoter, representative of active genes, and (**B**) for *Myoglobin* exon 2, representative of inactive genes, using an optimised level of 1 million cells, following increasing doses of UVB. The same assay was then applied to the analysis of T<>T levels in (**C**) *GAPDH* and (**D**) *Myoglobin* exon 2 genes, at 0, 6, 24 and 36 h after exposure to 0.1 J/cm^2^ SSR. Recovery, which represents the induction/repair of damage, was expressed as a percentage of the amount of immunoprecipitated DNA compared to the input DNA after DDIP-qPCR. The results are the mean ± SEM of three independent DDIP-qPCR experiments, **** *p* < 0.0001, ** *p* < 0.001.

The assay was then repeated to optimise the number of cells required to assess the UVB induction of CPD. The results revealed that when 1 million HaCaT cells were used, a good UVB dose-response was observed for both the *GAPDH* promoter (Figure 3A) and *Myoglobin* exon 2 (Figure 3B) loci, with a higher level of damage induction being noted at the active *GAPDH* promoter (Figure 3A) compared to the inactive *Myoglobin* exon 2 gene region (Figure 3B).

Following optimisation of the DDIP assay, the conditions were used to assess the induction and repair of CPDs in HaCaT cells following irradiation with SSR. Again, DDIP-qPCR was performed using *GAPDH* and *Myoglobin* exon 2 gene primers. The results demonstrated that SSR appears to preferentially induce T<>T in the active *GAPDH* gene, compared to the inactive *Myglobin* exon 2 gene regions (Figure 3C,D, respectively), confirming the results seen for naked DNA in Figure 2. For *GAPDH*, immediately after SSR irradiation, the percentage recovery of the immunoprecipitated sample to the input was 1.3%. This percentage decreased to 0.27% at 6 h, to 0.04% at 24 h, and to 0.028% at 36 h post-irradiation (Figure 3C). A markedly less rapid decrease was noted in the *Myoglobin* gene region, albeit with much less damage induced in the first place, and no noticeable repair over the first 6 h (Figure 3D). 

### 2.4. Nuclear and Mitochondrial Genome-Wide Mapping of T<>T Induction and Repair

**Figure 4:**
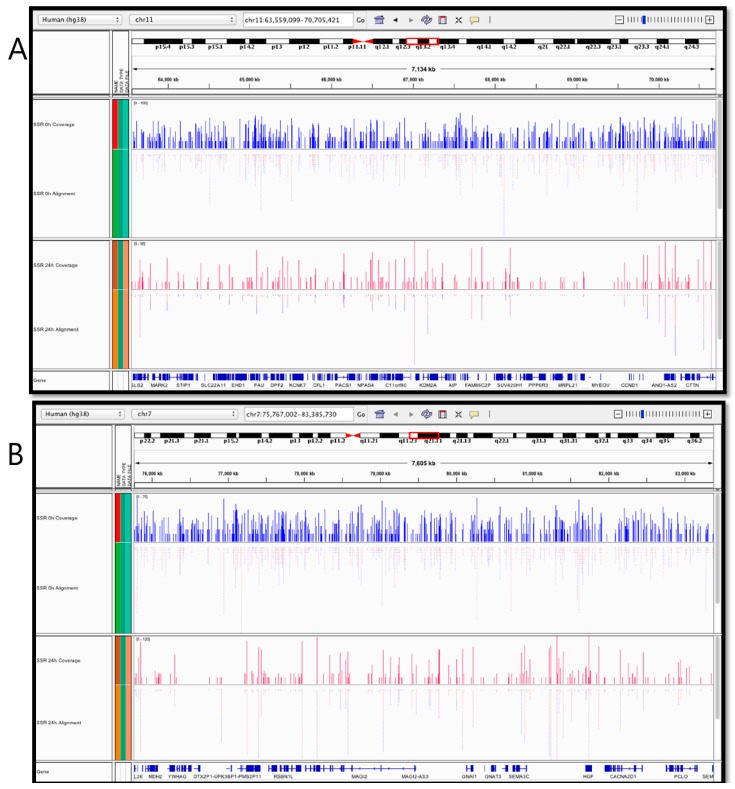
Screenshot of a representative Integrated Genomics Viewer [32] visualisation of the alignment of SSR-irradiated samples at 0 and 24 h post SSR exposure, mapped to human genome reference GRCh38 at (**A**) a 7134 kb region of chromosome 11, q13.2; and (**B**) a 7605 kb region of chromosome 7, q21.11. The blue (0 h, immediately after irradiation) and red (24 h after irradiation) coverage tracks correspond to the depth (number) of reads at each position. The pale pink and blue lines (below the solid blue and red lines in the upper and lower panels, respectively) represent the reads on both forward and reverse strands.

For the purposes of demonstrating the application of our DDIP-seq assay, our analyses focused specifically upon damage within gene regions. We identified the presence of damage in 13,680 genes in HaCaT cells immediately following irradiation with SSR. Representative results of the whole nuclear, genome-wide mapping of T<>T, to the human genome reference GRCh38, across a 7134 kb region of chromosome 11, q13.2, and a 7605 kb region chromosome 7, q21.11 are illustrated in Figure 4 (A and B, respectively). Figure 4A,B (upper panels, in blue) both illustrate a clear heterogeneous distribution of reads (damage), in terms of amount and location, induced immediately after irradiation (0 h) across both regions. Some regions clearly reveal higher levels of damage, with an absence of damage in other regions. At 24 h post-irradiation, the total levels of damage decreased (and the number of genes in which damage was detected had decreased to 10,822), and the number of locations lacking damage increased (Figure 4A,B, lower panels in red). Damage clearly persisted for at least 24 h in some regions, whereas in others it was fully repaired, which did not appear related to the initial, induced levels of damage. 

**Figure 5:**
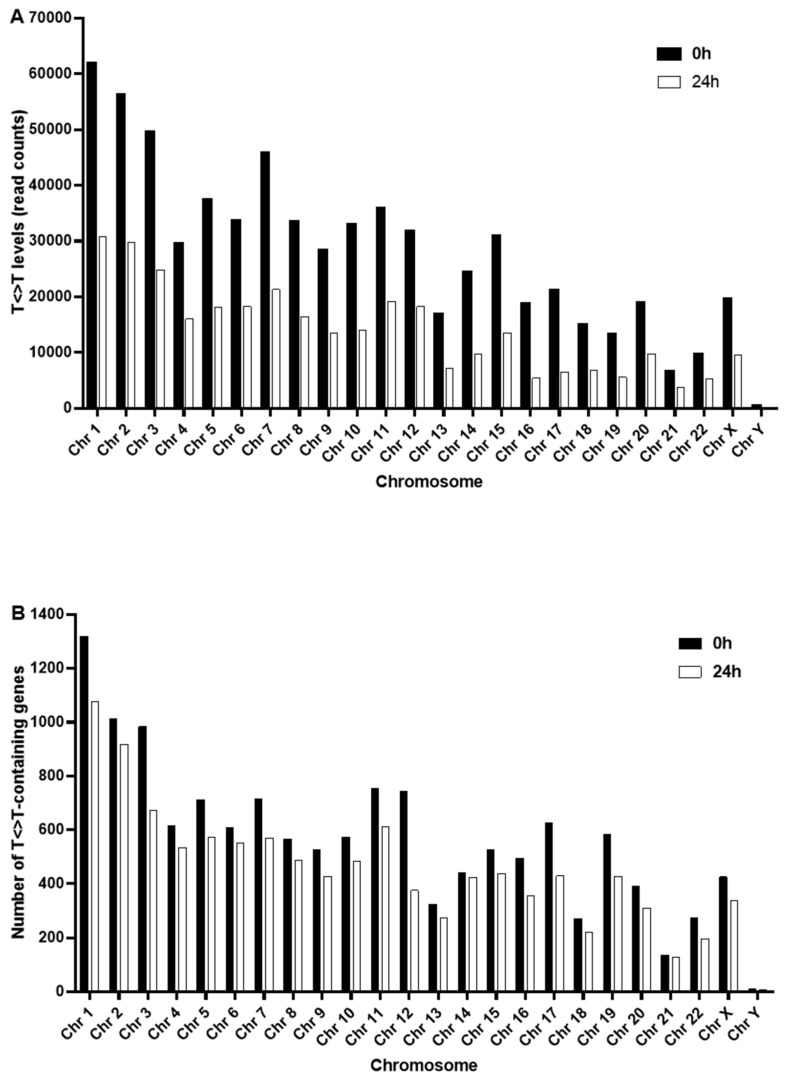
Distribution of T<>T levels (read counts) between chromosomes, in HaCaT cells irradiated with SSR, mapped to the human genome assembly GRCh38. (**A**) indicates the total level of T<>T per chromosome immediately post-SSR exposure (filled bars), and 24 h later (open bars). (**B**) indicates the number of damaged genes (i.e., containing one or more damaged moiety) per chromosome, immediately post-SSR exposure (filled bars), and 24 h later (open bars).

We extended this analysis to study the distribution of damage between chromosomes. Figure 5A illustrates the total levels of T<>T per chromosome, immediately after irradiation, and 24 h later. As might be expected for a directly damaging agent such as UVR, at this macro-scale, generally the total levels of T<>T per chromosome correlated with chromosome length. The exception to this was the X chromosome, which contained comparable levels of T<>T, before and after repair, to chromosome 20, despite being approximately 2.5 times longer. At this crude resolution, the amount of T<>T remaining after 24 h appeared to be proportional to initial levels of damage, representing a decrease of approximately 50% for each chromosome (Figure 5A). Expressing these data as number of T<>T-containing genes per chromosome (Figure 5B) revealed a similar distribution, for 0 h, to that seen in Figure 5A. However, there was less of a pronounced decrease in the number of T<>T-containing genes between 0 h and 24 h.

**Figure 6:**
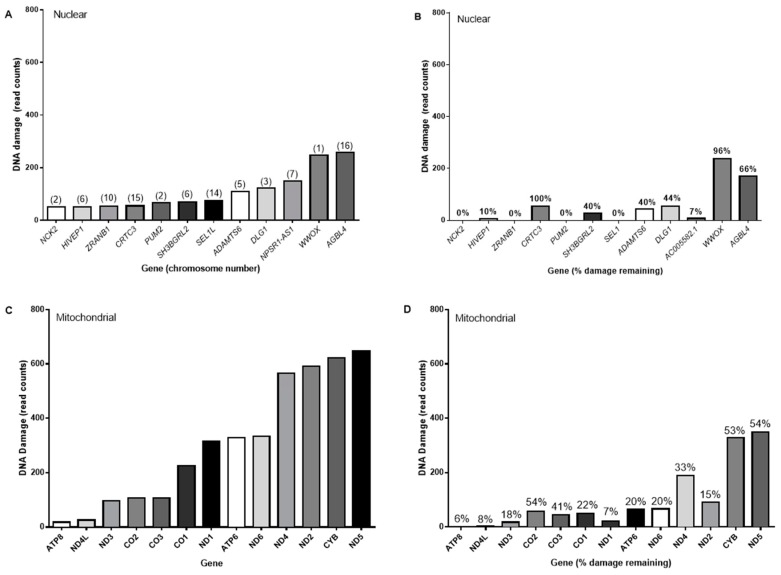
Levels of solar simulated radiation induced T<>T (read counts) in representative (**A**) nuclear, and (**C**) mitochondrial genomic regions, immediately and (**B** and **D**, respectively) 24 h after exposure to SSR. In Figure 6 (**A**), the corresponding chromosomal location is given in parentheses for the nuclear loci. Indicated in Figure 6 (**B**) and (**D**) is the percentage of damage remaining, relative to initial amounts in their corresponding figures (Figure 6A,C, respectively).

The heterogeneity in damage induction and repair noted in Figure 4. was confirmed by the detailed analysis of a smaller number of genes, as shown in Figure 6A, which illustrates differential sensitivities to damage formation, and rates of repair across a number of different nuclear genomic regions (Figure 6B). We also examined SSR-induced levels of T<>T at representative loci within the mitochondrial genome. Levels of damage were not uniformly distributed across the loci examined. Levels of damage tended to be higher at the mitochondrial loci (Figure 6C), compared the nuclear (Figure 6A), being as much as 2.5 greater, when comparing the most damaged loci in both genomes. Repair was more effective towards nuclear damage, with generally more damage persisting in the mitochondria, after 24 h. As was observed with nuclear damage, loss of T<>T in mitochondria did not appear to be influenced by initial levels.

**Figure 7:**
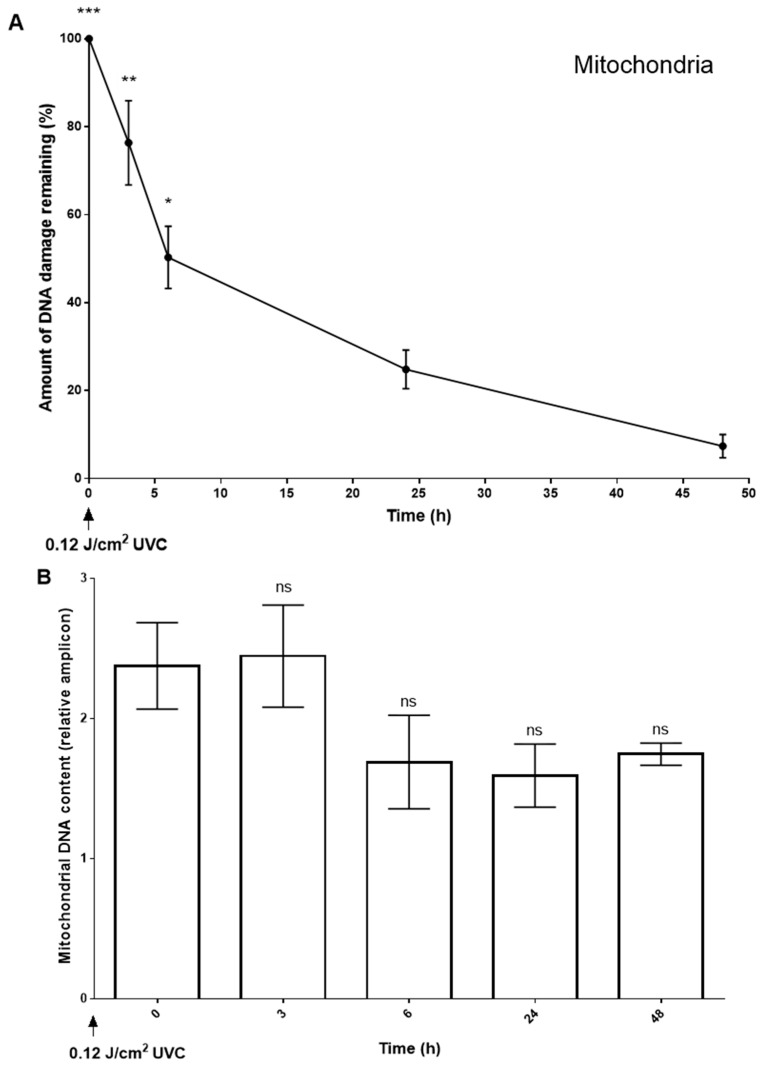
(**A**) The loss of solar simulated radiation induced-DNA damage (T<>T) from a representative region of the mitochondrial genome, determined by short-range qPCR. Points and bars represent the mean ± SEM of three independent experiments. *** *p* < 0.001, ** *p* < 0.01, and * *p* < 0.05, relative to an unirradiated sample. (**B**) Corresponding mitochondrial DNA content, from the experiment described in Figure 7A, determined by real-time qPCR. Bars represent the mean ± SEM of three independent experiments, ns = not significant, compared to the result for the zero h samples.

We used short-range qPCR as an alternative approach to further investigate the time-dependent loss of T<>T from the mitochondrial genome observed using DDIP-seq. This approach confirmed that levels of T<>T, in a 221 bp region spanning the *Cytb* and *ND6* genes, decreased significantly over a 48 h period post-irradiation (Figure 7A). Given that it is possible that the loss of T<>T was due to turnover of the genomes of damage-containing mitochondria, we therefore simultaneously evaluated mitochondrial DNA content in the irradiated HaCaTs. Although levels of mitochondrial DNA content appeared to decrease 6 h following irradiation, there were no significant differences in content between any of the timepoints (Figure 7B).

## 3. Discussion

There is a growing number of methods for studying the genome-wide induction of DNA damage and its repair [29,33,34]. Using our DDIP-seq method, we have mapped T<>T formation and repair across the entire nuclear and mitochondrial genomes. We noted that in the absence of repair (i.e., immediately after exposure), at high resolution, the induction of T<>T was distributed heterogeneously across the genome, presumably due to region-specific susceptibility to damage formation. Indeed, we showed that, following irradiation of extracted DNA, *GAPDH* appeared to be more intrinsically prone to damage formation than *Myoglobin* exon 2; suggesting the presence of possible conformational differences between the two genes that are present even in naked DNA, which render *GAPDH* more sensitive to damage formation. However, other recent studies describe a uniform induction of pyrimidine dimers [(6-4)PP and CPD)] [35] and cisplatin adducts [36]. At whole chromosome resolution, we noted similar results, but not at higher resolution. Indeed, a high-resolution examination of damage over representative 7134 or 7605 kb regions showed that the damage was heterogeneously distributed, confirming previous observations [7,37]. This finding was reiterated when we noted marked variation in the levels of damage induced in individual gene loci. Indeed, the distribution of damage, depending upon its source, appears to be determined by a variety of inter-related elements [38,39], e.g., nuclear organisation (e.g., greater damage in sites in proximity to the nuclear membrane) [30], nucleotide sequence [34,40], proximity of metal ions [41]; DNA-histone interactions, and epigenetic factors [42,43,44,45]. 

We also showed that repair is site-specific confirming the results of others, using genome-wide mapping techniques [27], and approaches targeted towards individual loci [46] [12,47]. It is not entirely clear whether heterogenous susceptibility, and hence damage distribution [48], or the distribution of repair activities [35,36], is primarily responsible for the steady-state distribution of damage across the genome. 

Although genome-wide mapping techniques are becoming used more frequently, little attention has been directed towards mitochondria, until one recent report [49]. These authors noted the presence of the DNA adduct 3-(2-deoxy-β-D-erythropentofuranosyl)pyrimido[1,2α]purin-10(3H)-one (M1dG) at roughly equal levels throughout the mitochondrial genome, with no specific sites of enrichment, compared to untreated cells. Here, we are the first to study the induction and loss of T<>T across the mitochondrial genome. Like the nuclear genome, we note an apparent non-random distribution of damage, evidenced by different levels of damage at representative gene loci, in contrast to the results for M1dG which the authors described as having no particular sites of accumulation [49]. Our findings are consistent with an assessment of ROS-induced DNA damage in specific coding regions of mitochondria, in which levels of damage differed across four sites (D-Loop, *COII/ATPase6/8*, *ND4*, *ND5*, and *ND1*) [50]. Unfortunately the authors did not report the repair of damage at these individual sites [50]. In contrast, a more recent report indicated that levels of oxidised purines appear to be the same across three mitochondrial DNA regions (D-loop, Ori-L, and *ND1*) [51].

Similar to the findings for M1dG, we noted that levels of T<>T were significantly higher in the mitochondrial DNA, compared to nuclear. We also noted a loss of T<>T from mtDNA with time, and observed that this was not equally distributed across the mitochondrial genome, with some loci targeted preferentially. Using an assay highly sensitive to the detection of thymine dimers, the loss of UVC-induced mitochondrial DNA damage has been reported previously and attributed to DNA repair [52]. In the case of M1dG, induced global levels of damage persisted in mtDNA for at least 24 h; however, a genome-wide analysis was not performed, unlike the present study, so whether or not the sequence-specific loss of M1dG occurred cannot be evaluated. 

In our study, mitochondrial levels of T<>T clearly decreased with time, determined by DDIP-seq, and confirmed qPCR. The term loss is used here, rather than repair, as it is widely considered that mitochondria have no NER pathway *per se*, for the removal of T<>T, although some NER-related proteins have been have been noted in the mitochondria, seemingly due to their association with the repair of oxidatively damaged DNA (reviewed in [53]). While alternative excision repair pathways exist in other species, to date, none have been reported in mammalian cells. It is possible that, in the absence of NER of T<>T or indeed M1dG, mitochondria with highly damaged DNA are degraded [54] or rescued by fusion with a mitochondrion with relatively undamaged DNA [55,56]. Were either the case, then one might expect the pattern of damage to remain the same at zero and 24 post-irradiation, with the same decrease in damage across all loci, but this was not the case. Furthermore, we studied the mtDNA content of UV irradiated cells across the time course of repair, and noted no significant changes in mtDNA content. Unless the production of new mitochondria and mitophagy was in equilibrium, these data suggest that T<>T can be actively removed from mtDNA, by unknown processes. From a cellular perspective, despite the ‘logistics’ of targeting NER proteins to the excess of mitochondrial genomes, compared to a singular nuclear genome, it might be more economical to repair bulky adducts, rather than generate new mitochondria. We are currently investigating this further. 

It is also worth noting the differences between global genome, and genome-wide assessments of damage and repair. We and others have demonstrated previously that the global genome repair of UVB-induced CPD is a lengthy process (t_1/2_ > 48 h) [57]. This is markedly different to the results with DDIP-seq, which revealed that some loci are fully repaired within 24 h of irradiation, whereas for others, up to 96% of the initial damage remains 24 h later. This indicates that, whilst informative, measurement for global genome levels of DNA damage and repair may not fully reflect events in specific regions of the genome; this in turn has consequences for follow-on biological effects, notably cell transformation and/or death. 

## 4. Conclusions

Like others, we have demonstrated that the induction and repair of damage is heterogenous in nuclear DNA, but importantly we have extended these investigations to include the mitochondrial genome and, for the first time, shown similar results as for the nuclear genome. These findings imply the presence of an, as yet, unidentified process for the removal of T<>T from mitochondrial DNA.

These data illustrate that genome-wide mapping adductomic approaches, such as DDIP-seq, provide the potential for developing a greater understanding of the formation and repair of damage, giving a more mechanistic insight into the link between DNA damage, repair, downstream events and disease, which is currently a “black box”.

## 5. Materials and Methods

### 5.1. Cell Culture

Human adult calcium temperature (HaCaT) cells are a spontaneously immortalised, human keratinocyte cell line. HaCaT cells were developed from a long-term culture of normal human skin keratinocytes at low calcium concentration and high temperature [58]. HaCaTs was kindly provided by Professor N.E. Fusenig (Deutsches Krebsforschungszentrum, Germany). HaCaT cells were cultured in Dulbecco’s Modified Eagle Medium/Nutrient Mixture F-12 (D-MEM/F-12) (1:1; Invitrogen, city, UK) with 10% fetal calf serum (FCS), 10% GlutaMAX™ I, and 1% sodium pyruvate in Nunclon™ culture flasks, at 37 °C in 5% CO_2_. 

### 5.2. Cell Preparation and Treatment

Cells at 80% confluence were irradiated with SSR, or UVB, or UVC on ice. Following irradiation, fresh growth medium was added, and the cells then incubated in a 5% CO_2_ incubator at 37 °C for different times to permit DNA repair, and/or evaluate viability. At each time point the cells were trypsinised and used in subsequent assays. 

The source of SSR was a SUNTEST^®^ CPS+ cabinet (Atlas, Mount Prospect, IL, USA), which was programmed to irradiate the cells with 0.1 J/cm^2^. This low dose of SSR is considered in the range of the erythemal dose (0.1 J/cm^2^–0.2 J/cm^2^) in Europe, according to TEMIS, http://www.temis.nl/uvradiation/UVdose.html). UVB irradiation was performed using a custom-made exposure cabinet (Hybec Ltd., Leicester, UK), as described previously [57]. For optimisation of some assay conditions, isolated DNA was irradiated at different doses of UVB (0.25, 0.5, 0.75 and 1 J/cm^2^). To study the loss of T<>T in mtDNA specifically, HaCaTs were cultured in Petri dishes, as above, and exposed to 0.12 J/cm^2^ UVC (as a model system for effectively inducing T<>T), before being returned to the cell culture incubator, for 3, 6, 24 and 48 h). At these specific time points, the Petri dishes containing cells were removed. DNA was extracted using a QIAamp DNA mini kit (Qiagen, Manchester, UK), and quantified using a NanoDrop One (Thermo Fisher Scientific, Waltham, MA, USA), prior to quantitative PCR analysis, as described below. For the ELISA, cells were exposed to 50 µM H_2_O_2_ for 30 min, on ice, (as described elsewhere [59]) before DNA was extracted, and ELISA performed, as referenced below.

### 5.3. Cell Viability

At 24, 48 and 72 h following SSR exposure, cell viability was assessed using the Human Annexin V-FITC Apoptosis Kit (Bender Medsystems, Vienna, Austria) as described in our previous study [57]. Briefly, cells were trypsinised and centrifuged at 300× *g* for 5 min, prior to resuspension in 5 mL of fresh media, transferred to FACS tubes, and centrifuged at 300× *g* for 5 min. The supernatant was discarded, and the pellet resuspended in 1 mL of Annexin buffer, followed by the addition of 4 µL of Annexin V–FITC conjugate, and incubated at room temperature for 10 min. Subsequently, 30 µL (0.05 mg/mL) propidium iodide was added, and the cells incubated at room temperature for 1 min. Finally, the cells were analysed by flow cytometry (FACScan flow cytometer, Becton Dickinson, Wokingham, UK) using CellQuest software (Becton Dickinson, Wokingham, UK).

### 5.4. DNA Extraction and Preparation

Following treatment, HaCaT cells were pelleted by centrifugation at 300× *g* for 5 min, and the supernatant discarded. The cell pellet was washed twice with PBS, resuspended in 10 mL of PBS and then centrifuged at 500× *g* for 5 min at 4 °C, and the supernatant discarded. The cell pellet was then resuspended in 500 μL of complete GenDNA Digestion buffer (5 μL of 200 × proteinase K added to 1 mL of GenDNA Digestion buffer) and incubated, at 50 °C for 18 h in a thermoshaker. DNA was extracted using the GenDNA module buffers (Diagenode MeDIP kit; Diagenode, Liège, Belgium), according to the manufacturer’s instructions. 

### 5.5. DNA Shearing

Genomic DNA was sheared by sonication (Soniprep 150, MSE, London, UK) to obtain 100–300 bp fragments. Sheared DNA samples were then analysed on 1% agarose electrophoresis gel.

### 5.6. DNA Immunoprecipitation (DDIP) Assay

The principle of the DDIP assay for 8-oxodG (OxiDIP-Seq) was first described by Amente et al. [25], and developed in house based upon the MeDIP kit from Diagenode. The kit was used as described by the manufacturer, but using an anti-thymine dimer antibody (clone KTM53, Kamiya Biomedical Company, Tukwila, WA, USA), with the DNA:antibody ratio optimised (1:1 μg/mL), as determined in the present study. For optimisation, DDIP-qPCR was performed using different ratios of DNA and anti-T<>T antibody (DNA:Ab): 1:0.1 μg/mL, 0.1:0.1 μg/mL and 1:1 μg/mL. Evidence for the specificity of the anti-T<>T Mab for T<>T, rather than ROS-induced DNA damage, was provided by ELISA [60], using DNA extracted from cells exposed to UVC, or H_2_O_2_.

The immunoprecipitated (IP) DNA sample incubation mix (without the DNA samples added) was prepared in a total volume of 65 μL for one immunoprecipitated (IP) and input (IN) sample as follows, using buffers supplied with the kit: Buffer A, 24 μL; Buffer B, 6 μL; water, 35 μL. Then, 65 μL of IP incubation mix and 10 μL of sheared DNA were added per tube, making the total volume per IP’d sample 75 μL. For the input (control; IN) sample, 13 μL of IP incubation mix and 2 μL of DNA were added, making the total volume 15 μL. The IN sample acts as an internal control and represents purified, total background genomic DNA taken prior to IP that does not undergo IP, but does undergo amplification by qPCR. The samples were then incubated at 95 °C for 3 min, quickly chilled on ice and then pulse microfuged for a short time at 4 °C. The IN samples were kept at 4 °C overnight. Throughout the experimental workflow for each IP DNA sample, a separate IN sample was also used. 

The anti-T<>T KTM53 antibody was added to each IP sample, and all contents were transferred to new tubes containing 20 μL of preblocked protein A/G beads. The tubes were then placed on a rotating wheel at 4 °C and incubated overnight. Next the IP samples were washed with 450 μL of ice-cold wash buffer-1 and placed on a rotating wheel for 5 min at 4 °C. After the incubation, the samples were centrifuged at 6000 rpm at 4 °C for 1 min, and the supernatant discarded. The bead pellets were washed again with 450 μL of ice-cold wash buffer-2 and -3 and twice with wash buffer-4.

The IN samples were treated in parallel with the IP samples from this point. The elution buffer was prepared (103.5 μL buffer D, 11.5 μL buffer E and 5 μL buffer F). Next, 120 μL of complete elution buffer was added to the bead pellets and IN samples. All the tubes were incubated in a thermo-shaker for 10 min at 65 °C (1000–1300 rpm). DNA was purified and eluted using a QIAquick^®^ PCR purification kit (Qiagen, Manchester, UK). Briefly, 600 μL of PB buffer was added to each tube. The tube’s contents were then transferred to QIAquick columns and centrifuged at 4000 rpm for 1 min. The filter column was washed with 700 μL of PE buffer and was then centrifuged at 4000 rpm for 1 min. The flow-through was then discarded. The filter columns were centrifuged at 13,000 rpm for 1 min and were eluted with 50 μL of EB. Then tubes were incubated at 50 °C for 5 min and centrifuged at 13,000 rpm for 1 min. The concentration of IP and IN samples were measured by a Qubit^®^ fluorimeter using a Qubit^®^ dsDNA HS Assay Kit, as directed by the manufacturer (Thermo Fisher Scientific, Altrincham, UK).

### 5.7. Removal of CPD Adducts Prior to PCR and Next-Generation Sequencing

CPD adducts are bulky and can block the DNA polymerase during the PCR amplification steps. The purified IP samples (25 μL) were all incubated with 1 μL of reagent from the PreCR DNA repair kit (New England Biolabs, city, UK) for 20 min at 37 °C, to remove the CPD prior to the analysis by qPCR. Then, 5 μL of the mixture were added to 15 μL of the master mix including the control primer. Then, the DNA samples were amplified as followed: 2 min at 95 °C, then 25 cycles as followed: 10 s at 95 °C, 30 s at 65 °C, and 1 min at 72 °C. After the PCR, DNA was purified using Qiaquick PCR purification kit (Qiagen, Manchester, UK) and eluted in 50 μL of elution buffer (Qiagen, Manchester, UK). 

### 5.8. Quantitative PCR Analysis

Following DIP, quantitative PCR (qPCR) was conducted to assess the success of the immunoprecipitation step. qPCR reactions were performed in duplicate in a final volume of 16 µL using the SensiMix™ SYBR^®^ Hi-ROX Kit (Bioline, London, UK), on a 7300 Real-Time PCR System (Applied Biosystems, Foster City, CA, USA). A total of 5 µL of IP and IN samples were used per reaction with 1 × SYBR green, and a mix of forward and reverse primer at a final concentration of 259 nM. Two primer sets were used for qPCR analysis; *GAPDH* and *Myoglobin* Exon 2 (Table 1). The PCR conditions included a 10 min denaturation step at 95 °C, followed by 40 cycles of 30 s at 95 °C, 30 s at 60 °C and 30 s at 72 °C.

### 5.9. MicroPlex Library Preparation™ Kit for Next-Generation Sequencing

The MicroPlex Library Preparation kit (Diagenode, Liège, Belgium) was used to prepare the indexed sequencing libraries for Illumina MiSeq next-generation sequencing. Briefly, template DNA was first repaired and blunt end molecules were generated using the manufacturer’s template preparation. Stem-loop adaptors were then ligated to the 5′ end of the genomic DNA and the 3′ ends of the genomic DNA were extended. The libraries were then amplified using Illumina-compatible index primers. Each single step was performed according to the manufacturer’s recommendations. 

The MicroPlex libraries were purified using AMPure^®^ XP beads (Beckman Coulter, High Wycombe, UK), and quantified by real-time qPCR using the KAPA Biosystems library quantification kit (Roche Diagnostics, Burgess Hill, UK) according to the manufacturer’s instructions. The indexed sequencing libraries were then pooled at equimolar concentration. The pool was spiked-in with 1% of PhiX and were sequenced on an Illumina MiSeq sequencer using 2 × 308 paired end sequencing. 

### 5.10. Bioinformatic Analysis of NGS Data

Raw sequencing fastq files were analysed using the bioinformatics pipeline, as follows. First, the data were preprocessed and quality control performed on the reads, anaTQC (http://goo.gl/6TUqD). Then, the adapter sequences were removed using Trimmomatic, and the read mapper Burrow–Wheeler Aligner (BWA; http://bio-bwa.sourceforge.net/) [61] was used for alignment. The reads from each sample were mapped to the human genome assembly GRCh38. Next, the mapped data (SAM files) were filtered, in BWA, using a mapping quality (MAPQ) score with a cut-off q30, to eliminate reads that mapped to more than one location in the genome, poor quality and erroneous alignments. Following the alignment, the resulting SAM files were sorted and indexed using SAMtools (http://www.htslib.org/) [62]. The Integrative Genomics Viewer (IGV) (http://www.broadinstitute.org/igv/) was used to visualise the mapped reads to the reference genome GRCh38. Next, the genes were identified per sample using the GENCODE tool to find gene annotations and to compare them at different time points following exposure to SSR. 

### 5.11. Quantification of mtDNA Damage

In order to further study the loss of T<>T from the mitochondrial genome noted by DDIP-seq, we performed “short range” qPCR of mtDNA damage in HaCaTs, using UVC as an effective source of T<>T. Using the method based on that of Santos et al. [63], total genomic DNA from UVC irradiated HaCaTs underwent PCR (Mastercycler Pro; Eppendorf, Hamburg, Germany) using primers specific for a 221 bp region spanning the *Cytb* and *ND6* genes, and using LongAmp Taq (New England Biolabs, Ipswich, MA, USA). The PCR products were quantified in a Synergy 2 microplate reader (BioTek, Winooski, VT, USA), based upon PicoGreen fluorescence. Lesion frequency/10 kb was calculated, according the formula reported elsewhere [64].

### 5.12. Quantification of mtDNA Content

Mitochondrial DNA content was evaluated via the analysis of a small region mtDNA (83 bp amplicon of D-loop), relative to a small region of a nuclear, single copy gene (93 bp amplicon of *beta 2 microglobulin*, *β2M*). The regions of interests were amplified using a real-time quantification PCR method, after modification and optimisation of the PCR conditions reported elsewhere [65,66,67]. The mitochondrial and nuclear DNA was amplified using Maxima SYBR Green qPCR Master Mix (2 x), and QuantStudio 3 (Applied Biosystems, Foster City, CA, USA). The relative amplification of mtDNA, and hence mtDNA content, was calculated using the 2−ΔΔCt method, as described in [67].

### 5.13. Statistical Analysis

All the experiments were conducted in triplicate and the results expressed as mean ± SEM, unless indicated otherwise. The statistical analysis was performed by one-way analysis of variance (ANOVA) test, using GraphPad Prism software v. 6.0. Significance limits were set at * *p* < 0.05, ** *p* < 0.01, *** *p* < 0.001 and **** *p* < 0.0001.

## Figures and Tables

**Table 1:** PCR primer sequences for target gene regions, performed by DIP-qPCR analysis.

***GAPDH* Primer Sequence**	
Forward	5′-GCCCCCGGTTTCTATAAATTG-3′
Reverse	5′-GTCGAACAGGAGGAGCAGAGA-3′
***Myoglobin* exon 2 Primer Sequence**	
Forward	5′-AAGTTTGACAAGTTCAAGCACCTG-3′
Reverse	5′-TGGCACCATGCTTCTTTAAGTC-3′

## Availability of Materials and Data

All materials, data and associated protocols are available to readers, subject to material transfer agreements.

## References

[1] Pfeifer G.P., Besaratinia A. (2009). Mutational spectra of human cancer. Hum. Genet..

[2] Kvam E., Tyrrell R.M. (1997). Induction of oxidative DNA base damage in human skin cells by UV and near visible radiation. Carcinogenesis.

[3] Premi S., Wallisch S., Mano C.M., Weiner A.B., Bacchiocchi A., Wakamatsu K., Bechara E.J., Halaban R., Douki T., Brash D.E. (2015). Chemiexcitation of melanin derivatives induces DNA photoproducts long after UV exposure. Science.

[4] Sancar A. (1996). DNA excision repair. Annu. Rev. Biochem..

[5] Wood R.D. (1997). Nucleotide excision repair in mammalian cells. J. Biol. Chem..

[6] Gale J.M., Nissen K.A., Smerdon M.J. (1987). UV-induced formation of pyrimidine dimers in nucleosome core DNA is strongly modulated with a period of 10.3 bases. Proc. Natl. Acad. Sci. USA.

[7] Zavala A.G., Morris R.T., Wyrick J.J., Smerdon M.J. (2014). High-resolution characterization of CPD hotspot formation in human fibroblasts. Nucleic Acids Res..

[8] Waters R., van Eijk P., Reed S. (2015). Histone modification and chromatin remodeling during NER. DNA Repair.

[9] Schick S., Fournier D., Thakurela S., Sahu S.K., Garding A., Tiwari V.K. (2015). Dynamics of chromatin accessibility and epigenetic state in response to UV damage. J. Cell Sci..

[10] Polak P., Lawrence M.S., Haugen E., Stoletzki N., Stojanov P., Thurman R.E., Garraway L.A., Mirkin S., Getz G., Stamatoyannopoulos J.A. (2014). Reduced local mutation density in regulatory DNA of cancer genomes is linked to DNA repair. Nat. Biotechnol..

[11] Tu Y., Tornaletti S., Pfeifer G.P. (1996). DNA repair domains within a human gene: Selective repair of sequences near the transcription initiation site. EMBO J..

[12] Ruven H.J., Seelen C.M., Lohman P.H., van Kranen H., van Zeeland A.A., Mullenders L.H. (1994). Strand-specific removal of cyclobutane pyrimidine dimers from the p53 gene in the epidermis of UVB-irradiated hairless mice. Oncogene.

[13] Branzei D., Foiani M. (2008). Regulation of DNA repair throughout the cell cycle. Nat. Rev. Mol. Cell Biol..

[14] Bohr V.A., Okumoto D.S., Ho L., Hanawalt P.C. (1986). Characterization of a DNA repair domain containing the dihydrofolate reductase gene in Chinese hamster ovary cells. J. Biol. Chem..

[15] You Y.H., Lee D.H., Yoon J.H., Nakajima S., Yasui A., Pfeifer G.P. (2001). Cyclobutane pyrimidine dimers are responsible for the vast majority of mutations induced by UVB irradiation in mammalian cells. J. Biol. Chem..

[16] Brash D.E., Haseltine W.A. (1982). UV-induced mutation hotspots occur at DNA damage hotspots. Nature.

[17] Doll R., Peto R. (1981). The causes of cancer: Quantitative estimates of avoidable risks of cancer in the United States today. J. Natl. Cancer Inst..

[18] Mao P., Brown A.J., Esaki S., Lockwood S., Poon G.M.K., Smerdon M.J., Roberts S.A., Wyrick J.J. (2018). ETS transcription factors induce a unique UV damage signature that drives recurrent mutagenesis in melanoma. Nat. Commun..

[19] Pfeifer G.P., Denissenko M.F., Tang M.S. (1998). PCR-based approaches to adduct analysis. Toxicol. Lett..

[20] Karakoula A., Evans M.D., Podmore I.D., Hutchinson P.E., Lunec J., Cooke M.S. (2003). Quantification of UVR-induced DNA damage: Global-versus gene-specific levels of thymine dimers. J. Immunol. Methods.

[21] Ohno M., Miura T., Furuichi M., Tominaga Y., Tsuchimoto D., Sakumi K., Nakabeppu Y. (2006). A genome-wide distribution of 8-oxoguanine correlates with the preferred regions for recombination and single nucleotide polymorphism in the human genome. Genome Res..

[22] Akatsuka S., Aung T.T., Dutta K.K., Jiang L., Lee W.H., Liu Y.T., Onuki J., Shirase T., Yamasaki K., Ochi H. (2006). Contrasting genome-wide distribution of 8-hydroxyguanine and acrolein-modified adenine during oxidative stress-induced renal carcinogenesis. Am. J. Pathol..

[23] Bryan D.S., Ransom M., Adane B., York K., Hesselberth J.R. (2014). High resolution mapping of modified DNA nucleobases using excision repair enzymes. Genome Res..

[24] Mao P., Smerdon M.J., Roberts S.A., Wyrick J.J. (2016). Chromosomal landscape of UV damage formation and repair at single-nucleotide resolution. Proc. Natl. Acad. Sci. USA.

[25] Amente S., Di Palo G., Scala G., Castrignano T., Gorini F., Cocozza S., Moresano A., Pucci P., Ma B., Stepanov I. (2019). Genome-wide mapping of 8-oxo-7,8-dihydro-2′-deoxyguanosine reveals accumulation of oxidatively-generated damage at DNA replication origins within transcribed long genes of mammalian cells. Nucleic Acids Res..

[26] Teng Y., Bennett M., Evans K.E., Zhuang-Jackson H., Higgs A., Reed S.H., Waters R. (2011). A novel method for the genome-wide high resolution analysis of DNA damage. Nucleic Acids Res..

[27] Hu J., Adar S., Selby C.P., Lieb J.D., Sancar A. (2015). Genome-wide analysis of human global and transcription-coupled excision repair of UV damage at single-nucleotide resolution. Genes Dev..

[28] Powell J.R., Bennett M.R., Evans K.E., Yu S., Webster R.M., Waters R., Skinner N., Reed S.H. (2015). 3D-DIP-Chip: A microarray-based method to measure genomic DNA damage. Sci. Rep..

[29] Crosetto N., Mitra A., Silva M.J., Bienko M., Dojer N., Wang Q., Karaca E., Chiarle R., Skrzypczak M., Ginalski K. (2013). Nucleotide-resolution DNA double-strand break mapping by next-generation sequencing. Nat. Methods.

[30] Yoshihara M., Jiang L., Akatsuka S., Suyama M., Toyokuni S. (2014). Genome-wide profiling of 8-oxoguanine reveals its association with spatial positioning in nucleus. DNA Res. Int. J. Rapid Publ. Rep. Genes Genomes.

[31] Powell J.R., Bennett M., Waters R., Skinner N., Reed S.H. (2013). Functional genome-wide analysis: A technical review, its developments and its relevance to cancer research. Recent Pat. DNA Gene Seq..

[32] Robinson J.T., Thorvaldsdottir H., Winckler W., Guttman M., Lander E.S., Getz G., Mesirov J.P. (2011). Integrative genomics viewer. Nat. Biotechnol..

[33] Grimaldi K.A., McGurk C.J., McHugh P.J., Hartley J.A. (2002). PCR-based methods for detecting DNA damage and its repair at the sub-gene and single nucleotide levels in cells. Mol. Biotechnol..

[34] Ding Y., Fleming A.M., Burrows C.J. (2017). Sequencing the Mouse Genome for the Oxidatively Modified Base 8-Oxo-7,8-dihydroguanine by OG-Seq. J. Am. Chem Soc..

[35] Hu J., Adebali O., Adar S., Sancar A. (2017). Dynamic maps of UV damage formation and repair for the human genome. Proc. Natl. Acad. Sci. USA.

[36] Hu J., Lieb J.D., Sancar A., Adar S. (2016). Cisplatin DNA damage and repair maps of the human genome at single-nucleotide resolution. Proc. Natl. Acad. Sci. USA.

[37] Yu S., Evans K., van Eijk P., Bennett M., Webster R.M., Leadbitter M., Teng Y., Waters R., Jackson S.P., Reed S.H. (2016). Global genome nucleotide excision repair is organized into domains that promote efficient DNA repair in chromatin. Genome Res..

[38] Cooke M.S., Evans M.D. (2010). Reactive Oxygen Species: From DNA Damage to Disease. Sci. Med..

[39] Poetsch A.R., Boulton S.J., Luscombe N.M. (2018). Genomic landscape of oxidative DNA damage and repair reveals regioselective protection from mutagenesis. Genome Biol..

[40] Mitchell D.L., Jen J., Cleaver J.E. (1992). Sequence specificity of cyclobutane pyrimidine dimers in DNA treated with solar (ultraviolet B) radiation. Nucleic Acids Res..

[41] Halliwell B., Aruoma O.I. (1991). DNA damage by oxygen-derived species. Its mechanism and measurement in mammalian systems. FEBS Lett..

[42] Carrell D.T. (2008). The clinical implementation of sperm chromosome aneuploidy testing: Pitfalls and promises. J. Androl..

[43] McLachlan R.I., O’Bryan M.K. (2010). Clinical Review#: State of the art for genetic testing of infertile men. J. Clin. Endocrinol. Metab..

[44] Hotaling J., Carrell D.T. (2014). Clinical genetic testing for male factor infertility: Current applications and future directions. Andrology.

[45] Stahl P.J., Masson P., Mielnik A., Marean M.B., Schlegel P.N., Paduch D.A. (2010). A decade of experience emphasizes that testing for Y microdeletions is essential in American men with azoospermia and severe oligozoospermia. Fertil. Steril..

[46] Bohr V.A., Smith C.A., Okumoto D.S., Hanawalt P.C. (1985). DNA repair in an active gene: Removal of pyrimidine dimers from the DHFR gene of CHO cells is much more efficient than in the genome overall. Cell.

[47] Denissenko M.F., Pao A., Tang M., Pfeifer G.P. (1996). Preferential formation of benzo[a]pyrene adducts at lung cancer mutational hotspots in P53. Science.

[48] Strand J.M., Scheffler K., Bjoras M., Eide L. (2014). The distribution of DNA damage is defined by region-specific susceptibility to DNA damage formation rather than repair differences. DNA Repair.

[49] Wauchope O.R., Mitchener M.M., Beavers W.N., Galligan J.J., Camarillo J.M., Sanders W.D., Kingsley P.J., Shim H.N., Blackwell T., Luong T. (2018). Oxidative stress increases M1dG, a major peroxidation-derived DNA adduct, in mitochondrial DNA. Nucleic Acids Res..

[50] Rothfuss O., Gasser T., Patenge N. (2010). Analysis of differential DNA damage in the mitochondrial genome employing a semi-long run real-time PCR approach. Nucleic Acids Res..

[51] Chimienti G., Picca A., Sirago G., Fracasso F., Calvani R., Bernabei R., Russo F., Carter C.S., Leeuwenburgh C., Pesce V. (2018). Increased TFAM binding to mtDNA damage hot spots is associated with mtDNA loss in aged rat heart. Free Radic. Biol. Med..

[52] Lehle S., Hildebrand D.G., Merz B., Malak P.N., Becker M.S., Schmezer P., Essmann F., Schulze-Osthoff K., Rothfuss O. (2014). LORD-Q: A long-run real-time PCR-based DNA-damage quantification method for nuclear and mitochondrial genome analysis. Nucleic Acids Res..

[53] Boesch P., Weber-Lotfi F., Ibrahim N., Tarasenko V., Cosset A., Paulus F., Lightowlers R.N., Dietrich A. (2011). DNA repair in organelles: Pathways, organization, regulation, relevance in disease and aging. Biochim. Biophys. Acta.

[54] Fakouri N.B., Hou Y., Demarest T.G., Christiansen L.S., Okur M.N., Mohanty J.G., Croteau D.L., Bohr V.A. (2018). Towards Understanding Genomic Instability, Mitochondrial Dysfunction and Aging. FEBS J..

[55] Vasileiou P.V.S., Mourouzis I., Pantos C. (2017). Principal Aspects Regarding the Maintenance of Mammalian Mitochondrial Genome Integrity. Int. J. Mol. Sci..

[56] Meyer J.N., Leuthner T.C., Luz A.L. (2017). Mitochondrial fusion, fission, and mitochondrial toxicity. Toxicology.

[57] Karbaschi M., Macip S., Mistry V., Abbas H.H., Delinassios G., Evans M.D., Young A.R., Cooke M.S. (2015). Rescue of cells from apoptosis increases repair in UVB exposed cells: Implications for the DNA damage response. Toxicol. Res..

[58] Boukamp P., Petrussevska R.T., Breitkreutz D., Hornung J., Markham A., Fusenig N.E. (1988). Normal keratinization in a spontaneously immortalized aneuploid human keratinocyte cell line. J. Cell Biol..

[59] Ji Y., Karbaschi M., Cooke M.S. (2019). Mycoplasma infection of cultured cells induces oxidative stress and attenuates cellular base excision repair activity. Mutat. Res..

[60] Cooke M.S., Podmore I.D., Mistry N., Evans M.D., Herbert K.E., Griffiths H.R., Lunec J. (2003). Immunochemical detection of UV-induced DNA damage and repair. J. Immunol. Methods.

[61] Li H., Durbin R. (2009). Fast and accurate short read alignment with Burrows-Wheeler transform. Bioinformatics.

[62] Li H., Handsaker B., Wysoker A., Fennell T., Ruan J., Homer N., Marth G., Abecasis G., Durbin R., Genome Project Data Processing S. (2009). The Sequence Alignment/Map format and SAMtools. Bioinformatics.

[63] Santos J.H., Meyer J.N., Mandavilli B.S., Van Houten B. (2006). Quantitative PCR-based measurement of nuclear and mitochondrial DNA damage and repair in mammalian cells. Methods Mol. Biol..

[64] Alrumaihi F.A. (2016). Assessment of UVR-Induced DNA Damage and Repair in Nuclear Genome versus Mitochondrial Genome. Ph.D. Thesis.

[65] Koch H., Wittern K.P., Bergemann J. (2001). In human keratinocytes the Common Deletion reflects donor variabilities rather than chronologic aging and can be induced by ultraviolet A irradiation. J. Investig. Dermatol..

[66] Malik A.N., Shahni R., Rodriguez-de-Ledesma A., Laftah A., Cunningham P. (2011). Mitochondrial DNA as a non-invasive biomarker: Accurate quantification using real time quantitative PCR without co-amplification of pseudogenes and dilution bias. Biochem. Biophys. Res. Commun..

[67] Hanna R., Crowther J.M., Bulsara P.A., Wang X., Moore D.J., Birch-Machin M.A. (2018). Optimised detection of mitochondrial DNA strand breaks. Mitochondrion.

